# Vitamin D Status in Preeclamptic and Non-preeclamptic Pregnant Women: A Case-Control Study in the North West of Iran

**DOI:** 10.15171/hpp.2015.022

**Published:** 2015-10-25

**Authors:** Bita Sadin, Bahram Pourghassem Gargari, Fatemeh Pourteymour Fard Tabrizi

**Affiliations:** ^1^Aras International Branch, Tabriz University of Medical Sciences, Tabriz, Iran; ^2^Nutrition Research Center, Department of Biochemistry and Diet Therapy, Tabriz University of Medical Sciences, Tabriz, Iran

**Keywords:** Vitamin D, 25-Hydroxyvitamin D, pre-eclampsia, pregnancy

## Abstract

**Background:** There are few studies on the vitamin D status in preeclamptic women. The objective of this case-control study was to determine vitamin D status, in preeclamptic women and compare it with that of healthy pregnant controls.

**Methods: ** Forty preeclamptic women, aged 18 to 45yr and forty age- and pregnancy weight-matched healthy controls participated in the study. Serum 25-hydroxy vitamin D (25(OH)D) levels were measured and its levels of <10, 10-30, 30-90 and >90 nanogram per milliliter (ng/ml) were considered as vitamin D deficiency, insufficiency, sufficiency, and toxicity, respectively.

**Results:** Sixty and forty percent of preeclamptic women were vitamin D deficient and insufficient, while in the control group they were 10% and 90%, respectively. No significant difference was found in the median intake of vitamin D between preeclamptic and non preeclamptic women (1.45 and 1.20µg/day respectively), but serum 25(OH)D concentration was significantly lower in preeclamptic cases compared to controls (10.09 ± 6.66 and 15.73 ± 5.85ng /ml respectively, P= 0.002) .

**Conclusion:** Vitamin D deficiency is common among preeclamptic and non-preeclamptic pregnant women in the north-west of Iran. Preeclampsia can cause decreasing of serum level of 25(OH)D.

## Introduction

The role of vitamin D in bone health was settled for many decades, while its role in pregnancy has been established 10-15 yr ago.^1^ Pre-eclampsia (PE) is a pregnancy-specific syndrome, which develops after 20 weeks of gestation. International Society for the study of Hypertension in Pregnancy (ISSHP) defines pre-eclampsia as: the existence of one or more of the following conditions: proteinuria, other maternal organ dysfunction: (renal, liver, neurological and hematological complications), and uteroplacental dysfunction during pregnancy.^2^ Preeclampsia can lead to eclampsia, which can put mother and her fetus at risk of death.^3^ Recently, the role of vitamin D on the health of the mother and fetus is being under more considration.^1^ Low status of vitamin D may result in low vitamin D activity and suppress the immune system and placental development.^4^ Therefore, a lack of vitamin D maybe involved in the pathophysiology of preeclampsia.^5^ The role of vitamin D in preeclampsia is related to the effect of vitamin D on renin-angiotensin system (RAS). Vitamin D is a negative endocrine regulator of RAS, which suppresses renin gene expression. Therefore, serum vitamin D levels are inversely associated with blood pressure and renin activity.^6^

Changes in vitamin D status and metabolism in preeclamptic patients has been reported.^7^ Unusual vitamin D status in preeclampsia and, consequently, considered the possibility of its deficiency is reported.^4^ However, the results of the literature review is controversial and vitamin D deficiency in preeclamptic patients has not been well demonstrated.Further studies on the issue are needed to clarify the link between vitamin D levels and preeclampsia.^7^

To the best of our knowledge, no study reported the vitamin D status of preeclamptic pregnant women in the north west of Iran, so the objective of this study was to determine vitamin D status, based on the serum 25(OH)D concentrations, in pregnant women with preeclampsia and compare it with that of healthy pregnant controls.

## Materials and Methods

### Study design

**Fig. 1:**
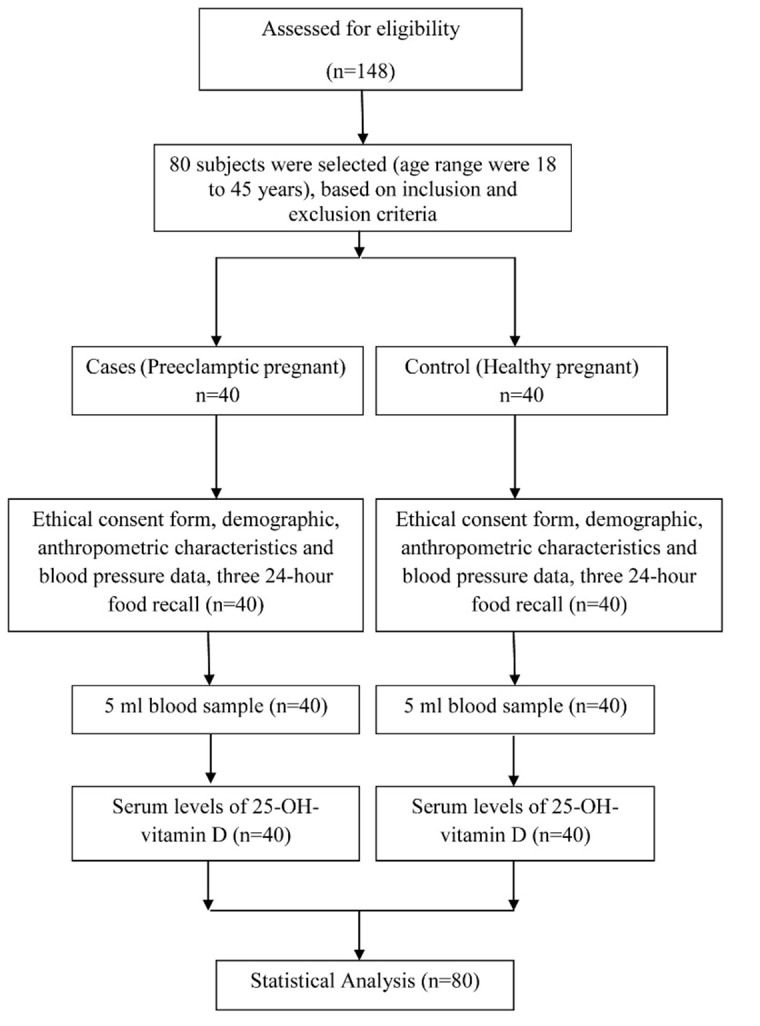
The flowchart of the study

This observational case-control study was done at the Al-Zahra Medical Teaching Center, (one of the main teaching hospitals of Tabriz University of Medical Sciences in North West of Iran) from early April 2013 to late January 2014. To determine the sample size, basic data including corrected odds ratio(OR) = 2.16, were obtained from the study of Baker et al.^4^ By using G-Power software and taking 95% confidence interval and 80% power, the sample size was estimated as 40 subjects in each group. The subjects agreed to participate in the survey after receiving sufficient explanation on the purpose and content of research. Forty preeclamptic and forty normal pregnant women as controls, seeking treatment at the Department of Obstetrics and Gynecology of Al-Zahra Hospital in Tabriz, Iran, were selected. The control group included healthy pregnant women matched for pregnancy weight, pregnancy age and kind of supplement intake. All subjects received similar multivitamin/mineral capsule. The participants’ age range was18 to 45 years (Figure 1).

### Baseline Definitions and Measurements

Preeclampsia was diagnosed if there was a blood pressure (BP) of 140/90 mmHg or greater, measured twice ≥ 6 hours apart, and consistent proteinuria of 300 mg/d or more after the 20th gestational weeks.^8^

Exclusion criteria were chronic hypertension or history of hypertension, pre-existing diabetes, history of renal disease, multiple pregnancies, entry to the delivery phase, current steroid therapy, and use of diuretics, β-blockers and non-steroidal anti-inflammatory drugs. Following an initial screening for inclusion and exclusion criteria and after obtaining informed written consent, qualified volunteers were scheduled for testing.

At the beginning of the study, venous blood sample (5cc) was taken, and immediately centrifuged. Serum was separated from whole blood, transferred to micro tube, and stored at −70 °C until biochemical assays were performed. Blood samples were obtained between 8:00 a.m. and 10:00 a.m. after overnight fasting. During the same visits, all patients underwent anthropometric measurements, and dietary assessments for three days (including 2-week days and 1 weekend) using a 24-h dietary recall.

Dietary data were analyzed by Nutritionist IV software. Information on anthropometric measurements and dietary intakes, were collected by trained personnel. Weight was measured without shoes and in light clothing using a digital scale with a precision of 0.1 kg (SECA 707; HH, Modena, Italy). Standing height was measured without shoes, using a stadiometer to the nearest 0.1 cm precision. Body mass index (BMI) was calculated by dividing body weight (kilograms) by height squared (meters). The pregnancy weight gain was calculated from pre pregnancy weight and current measured weight. The systolic and diastolic blood pressure (SBP and DBP) (5 min seated rest, mean of two readings) were measured with a mercury sphygmomanometer.

### Biochemical Measurements 

A competitive chemiluminescent Immunoassay system on the automated LIAISON ® analyzer (Stillwater, MN) was used for the measurement of serum 25- hydroxyvitamin D [25(OH)D] concentration (DiaSorin, Saluggia, Italy). This method has 100% specificity for both 25(OH) vitamin D_3_ and 25(OH) vitamin D_2_. This assay is a two-step procedure. First, 25(OH)D and other hydroxylated metabolites are rapidly extracted from serum using acetonitrile. The extracted sample is then assayed using an antibody with specificity to 25(OH)D. The intra-assay an inter assay coefficient of variation (CV) of the method for vitamin D were less than 10%. Serum 25(OH)D levels of <10, 10-30, 30-90 and >90 ng/ml were considered as vitamin D deficiency, insufficiency, sufficiency, and toxicity, respectively.^9,10^

### Ethical considerations

Informed consent was taken from patients. The study protocol was approved by the Ethics Committee of the Tabriz University of Medical Sciences, Tabriz, Iran.

### Statistical Analysis

Normality analyses were made by the Kolmogorov–Smirnov test. Data showing a normal distribution were presented as mean value ± standard deviation (SD) and data without a normal distribution were reported as median. The socio demographic characteristics of the groups were evaluated using the chi-square test. The independent sample *t*-test or Mann- Whitney U test were used to compare the groups in a parametric or nonparametric way. Multiple logistic regression was used for calculating adjusted odds ratio. Statistical analyses were performed using SPSS 16.0. A value of *P*<0.05 was considered as significant in all statistical analyses.

## Results

No differences were found between the patient and the control groups with respect to the latitude of residence and kind of supplement intake. All subjects were resident of North West of Iran and received similar multivitamin/mineral. General characteristics of the study population are presented in Table 1. The patient and the control groups were matched according to pregnancy weight, pregnancy age and kind (type and dose) of supplement intake; thus, there were no significant differences in these characteristics between the two groups (*P*> 0.05) (Table 2).

**Table 1:** Demographics and dietary variables of the studied subjects

**Variables**		**Preeclamptic women** **(n= 40)**	**Non Preeclamptic pregnant women (n=40)**	***P***
Gestational age; mean (SD)(wk)		30.95 (4.26)	31.08 (4.13)	0.91^§^
Maternal Age; mean (SD) (yr)		32.05 (6.00)	28.20 (6.00)	0.006^§^
Occupation (%)	Housekeeper	50	50	1.00^ҙ^
	Employee	50	50		
Education (%)	Illiterate	10	5	0.087^ҙ^
	Incomplete secondary	45	60	
	Diploma	32.5	35	
	University	12.5	0	
Habitation (%)	Rural	62.5	35	0.014^ҙ^
	Urban	37.5	65	
Family history of preeclampsia (%)		15	7.5	0.288^ҙ^
Smoking (%)		20	12.5	0.363^ҙ^
Energy intake (Kcal/day)		1822 (353)	1751 (329)	0.356^§^
Carbohydrate intake (g/day)		276 (60)	262 (55)	0.273^§^
Protein intake (g/day)		83.6(15.8)	80.4 (17.1)	0.383^§^
Fat intake (g/day)		44.7 (11.4)	44.4 (10.1)	0.913^§^
Vitamin D intake (µg/day)		1.45 (1.06-2.07)	1.20 (1.00-1.53)	0.077^*^

Number of subjects //§: Independent T-test was used to compare mean differences (Mean (SD)) among groups. ҙ: Chi-Square test was used to compare the two groups with regards to sociodemographic (categorical) variables. :*Dietary vitamin D intake was reported in median (interquartile ranges) and 25^th^-75^th^ percentiles. Mann-Whitney U test was utilized to compare dietary vitamin D intake difference between two groups.

Totally, 60% of the preeclamptic women were vitamin D-deficient, as reflected by the number of persons with serum 25(OH)D levels below 10 ng/ml and 40% were vitamin D insufficient, as reflected by the number of persons with serum 25(OH)D levels between 10 ng/ml to 30 ng/ml. In control group 10% and 90% were vitamin D deficient and vitamin D insufficient, respectively. No differences were found between the patient and the control groups with respect to weight, height, body mass index, or dietary energy, protein and vitamin D intakes (*P*> 0.05). Dietary intake data showed that, as compared to Recommended Dietary Allowances (RDA), 100% of the patients and the controls had a low vitamin D intake (data not shown). Data, as presented in Table 3, showed that mean serum concentration of 25(OH)D  of preeclamptic women were significantly lower than that of healthy controls (*P*=0.002).

**Table 2:** Basic anthropometric characteristics of the studied subjects

**Variables**	**Preeclamptic women** **(n= 40)** **Mean (SD)**	**Non preeclamptic pregnant women(n=40)** **Mean (SD)**	***P*** -value*
Pre-pregnancy weight (kg)	73.15 (12.02)	74.37 (12.56)	0.657
Pre-pregnancy BMI (kg/m^2^)	28.89 (5.43)	30.03 (4.67)	0.320
Pregnancy weight (kg)	84.48 (13.62)	83.52 (13.54)	0.752
Pregnancy BMI (kg/m^2^)	33.49 (6.06)	33.72 (4.96)	0.854
Weight gain during pregnancy(kg)	11.25(4.37)	8.90(4.52)	0.021

*.Independent sample *t*-test// BMI: body mass index

The mean values of SBP and DBP were significantly higher in the preeclamptic subjects compared to the control ones (*P* < 0.001, Table 3). Maternal 25(OH)D concentration less than 10 ng/ml was associated with a 15-fold increase in the odds ratio of preeclampsia, compared to 25(OH)D concentration equal to or greater than 10 ng/ml (Table 4).

**Table 3:** Clinical characteristics of the studied subjects

**Variables**	**Preeclamptic women** **(n= 40)** **Mean (SD)**	**Non preeclamptic pregnant women(n=40)** **Mean (SD)**	**Unadjusted** *** P*** -value*	**Adjusted ** ***P*** **-value**
SBP (mmHg)	151.50(10.99)	109.63 (12.21)	0.001	<0.001
DBP (mmHg)	97.25 (8.16)	71.50 (11.33)	0.001	<0.001
25(OH)-vitamin D(ng/ml)	10.09 (6.60)	15.73 (5.85)	0.001	0.002

*.Independent sample *t*-test 25(OH)D: 25-hydroxy vitamin D, SBP: Systolic blood pressure, DBP: Diastolic blood pressure.

**Table 4:** Unadjusted and adjusted Odds ratio for preeclampsia based on vitamin D status in the studied subjects

**Serum 25(OH)D (ng/ml)**	**Preeclamptic** **Women** **(n=40)**	**Non preeclamptic pregnant women (n=40)**	**Unadjusted odds ratio (CI 95%)**	***P*** **-value**	Adjusted odds ratio (CI 95%)*	***P*** **-value**
≥10	16	36	1.00 (Reference)	-	1.00 (Reference)	-
<10	24	4	13.50 (4.02-45.33)	<0.001	14.98(4.01-55.95)	<0.001*

*. Adjusted for body mass index, preeclampsia history, smoking, and maternal age.

## Discussion

Maternal vitamin D deficiency is a widespread public health problem that is prevalent during pregnancy.^11^ Vitamin D acts in various physiological processes as vascular health, immune function, metabolism and function of the placenta.^1^ Vitamin D has wide functions during pregnancy, including the effects on placental function and inflammatory response.^12^ Our study showed lower vitamin D levels in the preeclamptic pregnant women than the healthy pregnant controls. Based on the demographic data, there were no significant differences in the latitude of residency, amount of vitamin D intake between the two groups.

In this study, significant difference was found in serum 25(OH)D levels between pregnant women with preeclampsia and healthy pregnant controls. Our results confirm the findings of previous studies, in which lower serum 25(OH)D concentration was reported in patients with preeclampsia–compared to healthy pregnant controls.^7,13^ The relation between preeclampsia and vitamin D has been observed as early as 2007by Bodnar et al., who claimed that, a 50 nmol/l decrease in 25(OH)D concentration doubles the risk of preeclampsia in pregnant women.^14^ This relation between preeclampsia and vitamin D was documented earlier that vitamin D may influence fetal growth through placental mechanisms.^15^

Our results showed that maternal 25(OH)D concentration less than 10 ng/ml was associated with a 15-fold increase in the odds ratio of preeclampsia (adjusted OR, 14.98; 95% CI,4.01–55.95), compared to 25(OH)D concentration equal to or greater than 10 ng/ml. This association was significant and accordant with findings of recent three systematic reviews and meta-analysis of observational studies, which found that maternal risk of preeclampsia has been associated with low maternal circulating levels of25(OH)D.^7,13,16^ A large cohort study,^17^ consisted of over 23,000 nulliparous women, showed that the women who had vitamin D intake of 15-20 μg/day had 0.76 (95% confidence interval: 0.60-0.95) odds ratio of preeclampsia compared to those who had less than 5 μg/day. However, in a study no effect of vitamin D intake on preeclampsia occurrence was found.^18^ Two studies have evaluated longitudinal vitamin D levels in pregnant women who developed preeclampsia compared with those who did not; both failed to detect any relation between preeclampsia and vitamin D level.^19,20^ Possible explanations for these inconsistent findings can be related to the differences in dietary intakes of vitamin D, dietary intakes of other nutrients, vitamin D status of the subjects and genetically differences in vitamin D metabolism. Our study findings support the hypothesis that reduced level of serum vitamin D is related to the elevated risk of preeclampsia. The hypothesis that maternal vitamin D status could modify the risk of preeclampsia is biologically probable. Vitamin D can impact on maternal immune system responses to the fetus.^1,4,5^ Vitamin D deficiency associated with endothelial dysfunction due to inflammation.^21^ Thus, the anti-inflammatory properties of vitamin D have been emphasized.^22^

Vitamin D through several mechanisms can be considered as an important factor in the prevention ofpreeclampsia.^23^ Proinflammatory response, increase oxidative stress and endothelial dysfunction that characterize preeclampsia can arise from maternal vitamin D deficiency. In addition, vitamin D affects the expression of genes responsible for trophoblast invasion and angiogenesis, both of which are crucial for implantation.^22,24^ This process might be an important factor in the pathophysiology of preeclampsia.^25^Immune and vascular defects can lead to poor placental invasion, that leads to the release of placental-derived vasoconstrictor factors and consequent maternal hypertension and proteinuria.^26^ Vitamin D receptors on the heart and blood vessels suggest vitamin D has a cardio-protective effect, and it can influence endothelial and vascular smooth muscle cell function as well as controlling inflammation and affecting the regulation of blood pressure through influences on the renin-angiotensin-aldosterone system.^6^ Vitamin D is one of the most potent hormones which suppress the rennin-angiotensin system and thus regulate blood pressure. Vitamin D through influences on angiogenesis can reduce preeclampsia risk.^6,27^ This claim is supported by the stimulatory effects of vitamin D on the expression of endothelial growth factor in vascular smooth-muscle cells.^22,28^

Our study has several strengths. The study is the first one in the North West of Iran that has investigated the association between preeclampsia and vitamin D statues. We excluded women with chronic medical diseases such as chronic hypertension and diabetes. The mentioned chronic medical diseases can interference with preeclampsia. Our investigation has some limitations. Firstly, a relatively small number of participants were recruited for our study. Secondly, our observational data are hypothesis generating, and these findings should be considered preliminary. The case-control design of this study did not clearly elucidate the cause-and-effect on results. Thirdly, vitamin D input from sunlight, has not been evaluated quantitatively.

## Conclusion

Our results showed inappropriate vitamin D status, based on the serum 25(OH)D concentration, in preeclamptic pregnant women compared to the healthy controls. Since the study showed that lower levels of serum vitamin D could increase the risk of preeclampsia, increasing intake of vitamin D, particularly in those with clearly abnormal lower serum levels, may protect against preeclampsia, but this has yet to be proven in clinical trials.

## Acknowledgments

The authors would thank to the Nutrition Research Center and Department of Biochemistry and Diet Therapy, Tabriz University of Medical Sciences and the personnel of AL-Zahra Hospital, Tabriz, Iran. The study was written based on the data for MS thesis in Nutrition in Aras International Branch, Tabriz University of Medical Sciences.

## Competing Interests

The authors declare that there is no conflict of interest.
